# C1q A08 Is a Half-Cryptic Epitope of Anti-C1q A08 Antibodies in Lupus Nephritis and Important for the Activation of Complement Classical Pathway

**DOI:** 10.3389/fimmu.2020.00848

**Published:** 2020-05-27

**Authors:** Wen-Jun Wu, Ying Tan, Xiao-Ling Liu, Feng Yu, Ming-Hui Zhao

**Affiliations:** ^1^School of Life Science, Tsinghua University, Beijing, China; ^2^Renal Division, Department of Medicine, Peking University First Hospital, Beijing, China; ^3^Institute of Nephrology, Peking University, Beijing, China; ^4^Key Laboratory of Renal Disease, Ministry of Health of China, Beijing, China; ^5^Key Laboratory of Chronic Kidney Disease Prevention and Treatment, Ministry of Education of China, Beijing, China; ^6^Tsinghua-Peking Center for Life Sciences, Beijing, China; ^7^Department of Nephrology, Peking University International Hospital, Beijing, China

**Keywords:** lupus nephritis, C1q A08, half-cryptic epitope, complement classical pathway, epitope mapping

## Abstract

To investigate the fine epitope(s) of anti-C1q A08 antibodies and their roles in complement activation in lupus nephritis, C1q A08 and related peptides with various amino acid sequences around A08 were synthesized. Anti-C1q A08 antibodies from 10 lupus nephritis patients were purified from plasmapheresis samples, and four monoclonal antibodies against C1q A08 were screened and identified from mouse hybridoma cells, to study the fine epitope(s) of C1q A08 using ELISA and Biolayer Interferometry (BLI). The biofunction of anti-C1q A08 antibodies for complement classical pathway activation was investigated by C3 activation assay. Anti-C1q A08 antibodies and anti-C1q antibodies were also detected in the sera of female BALB/C mice immunized by C1q A08 peptides. None of the anti-C1q A08 antibodies, which were affinity purified from the 10 lupus nephritis patients, could bind intact C1q coated on microtitre plates, neither could the anti-C1q antibodies bind to C1q A08 peptides coupled on resin, indicating that the human anti-C1q antibodies and anti-C1q A08 antibodies may recognize different epitopes of C1q. One of the four C1q A08 mAbs (32-4) bound to the six amino acids of N-terminus of C1q A08, while another C1q A08 mAb (17-9) bound to eight or 10 amino acids of C-terminus of A08. The third and fourth C1q A08 mAb (1A12 and 4B11) bound to the whole sequence of A08. Only 32-4 mAb bound to the intact C1q coating on an ELISA plate, whereas 17-9 mAb, 1A12 mAb, and 4B11 mAb could not. However, using a BLI assay, 17-9 mAb, 1A12 mAb, and 4B11 mAb, but not 32-4 mAb, could bind to intact C1q. Furthermore, 1A12 mAb and 4B11 mAb, but not 32-4 and 17-9 mAb, could inhibit the activation of complement classical pathway. Anti-C1q A08 antibodies were detected in all the female BALB/C mice in the experimental group but not in the control group. Two out of six in the experimental group developed anti-C1q antibodies. C1q A08 is a half-cryptic epitope of C1q involving N-terminal six amino acids of C1q A08, and this is important to the activation of a complement classical pathway, and some anti-C1q A08 antibodies were able to prevent this process. Epitope spreading of C1q occurred in the mice immunized with C1q A08 peptides.

**Graphical Abstract:**
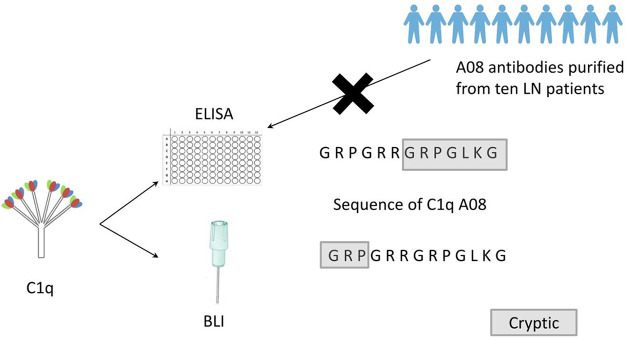
Clq A08 is a half-cryptic epitope. None of the anti-Clq A08 antibodies affinity purified from the ten lupus nephritis patients could bind intact Clq coated on microtitre plates. Clq A08 is important in activation of complement classical pathway and some anti-Clq A08 antibodies were able to prevent this process. Epitope spreading of Clq occurred in mice immunized with Clq A08 peptide.

## Introduction

Systemic lupus erythematosus (SLE) is an autoimmune disease characterized by breach of immune tolerance with an overproduction of various autoantibodies, such as anti-dsDNA antibodies, anti-Smith antibodies, and anti-C1q antibodies (1). SLE patients can develop several complications and target organ inflammation, of which lupus nephritis is the most crucial risk factor of morbidity and mortality. Almost all SLE patients exhibit deposition of immune complexes in glomeruli, 40–60% of which develop clinical lupus nephritis (2). The complement system plays an important role in the clearance of apoptotic cells, which release numerous cellular substances, such as dsDNA and proteins (3). The complement classical pathway is of major interest in lupus nephritis research, and C1q is the first protein in this classical pathway whose deficiency indicates a much higher risk of developing SLE. Interestingly, few patients exhibit C1q gene mutation, but anti-C1q antibodies have been found in more than 50% of lupus nephritis patients. Several studies have found an association between anti-C1q antibodies and disease activity in lupus nephritis (4–6), but its role in the pathogenesis of lupus nephritis still remains to be elucidated.

C1q, an ultra large protein with a molecular weight of about 460 kDa, is composed of six A chains, six B chains, and six C chains. The A chain is covalently linked to the B chain, while the C chain is non-covalently linked to the AB dimer in one ABC strand and is also covalently linked to the C chain of another ABC strand to form an ABC-CBA doublet (7). Each chain includes an N-terminal collagen-like region (CLR), which is responsible for mediating immune mechanisms, and a C-terminal globular head region, which is responsible for recognizing target ligands, such as an immune complex and bacterial and viral surface proteins (8). C1q is an important linker protein between the innate immune system and adaptive immune system through binding to antigen–antibody immune complexes to activate a complement classical pathway. In SLE, the generation of autoantibodies along with the deposition of immune complexes causes chronic inflammation and tissue injury, where C1q acts a significant role in clearing such immune complexes and apoptotic cells (9–11).

Importantly, Vanhecke et al. used anti-C1q antibodies derived from SLE patients in a microarray-based scan to identify the B-cell epitope of C1q, and found that C1q A08 (C1q A15-27: GRPGRRGRPGLKG) is the most important epitope of C1q (12). It was also found that binding of C1q A08 is correlated to bindings of C1q for the same sera, where C1q A08 seemed to be an exposed epitope on C1q. Our recent study, based on a large Chinese cohort, further confirmed that C1q A08 antibodies are better than antibodies against intact C1q in correlating with lupus nephritis activity as well as predicting renal prognosis (13).

The sequence of C1q A08 contains four arginine residues and one lysine residue, which is strongly positively charged. Prior studies have shown that C1q A14-26, with only one amino acid shift as compared to C1q A08 peptides, can bind some ligands, such as DNA (14), CRP (15, 16), fibronectin (17), LPS (18, 19), vWF (20), and amyloid P (21) as well as advanced glycan end products (22), indicating that C1q A08 may be an important functional sequence. It was believed that characterizing C1q A08 in greater detail would help better understand the biology of C1q in physiological and pathological conditions, particularly in the pathogenesis of lupus nephritis.

Therefore, anti-C1q A08 antibodies from lupus nephritis patients were purified, and four monoclonal mouse anti-human C1q A08 antibodies were screened. Various conformational epitopes and biofunctions of the anti-C1q A08 antibodies were further investigated *in vitro*.

## Results

### Binding of C1q to Anti-C1q A08 Antibodies Purified From Plasma Samples of Lupus Nephritis

**Figure 1:**
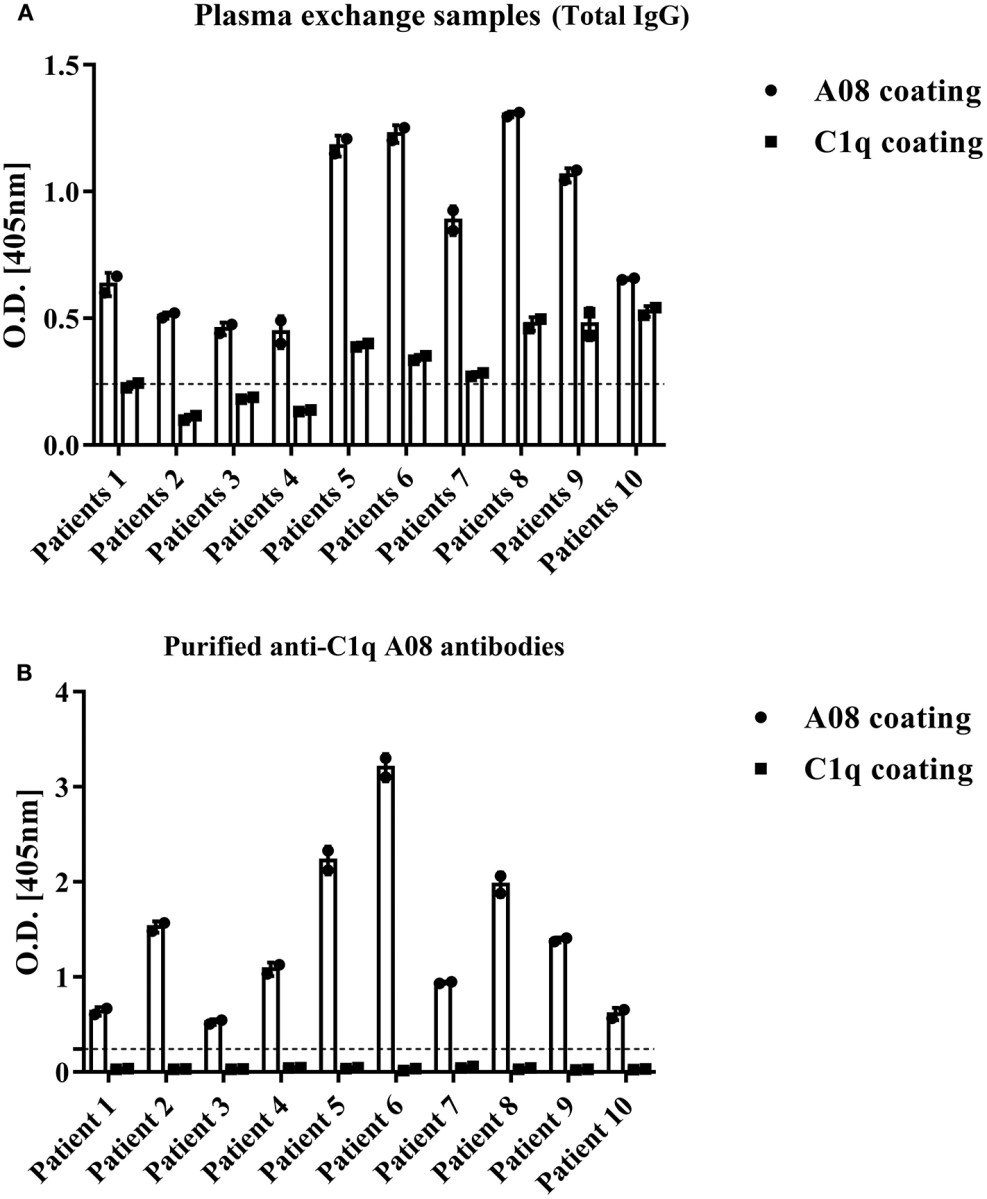
Binding property of anti-C1q A08 antibodies purified from plasma exchange of lupus nephritis patients. **(A)** Anti-C1q A08 antibodies and anti-C1q antibodies measured with ELISA method. **(B)** Binding of anti-C1q A08 antibodies purified from the plasma exchange with C1q A08 or C1q in ELISA.

To study the fine epitope(s) of anti-C1q A08 antibodies, plasma exchange samples from 10 lupus nephritis patients, who were positive for anti-C1q A08 antibodies, were used to isolate and purify anti-C1q A08 antibodies (Figure 1A). Total IgG was purified using a Protein G column, and anti-C1q A08 antibodies were isolated from total IgG using Pabpur SulfoLink beads coupling with GRPGRRGRPGLKGC (C1q A08 peptides fused with cysteine). All the anti-C1q A08 antibodies from the 10 lupus nephritis patients were bound to A08 but not intact C1q (Figure 1B).

### Mapping of Binding Site of C1q Using Monoclonal A08 Antibodies Derived From Hybridoma Cells

**Figure 2:**
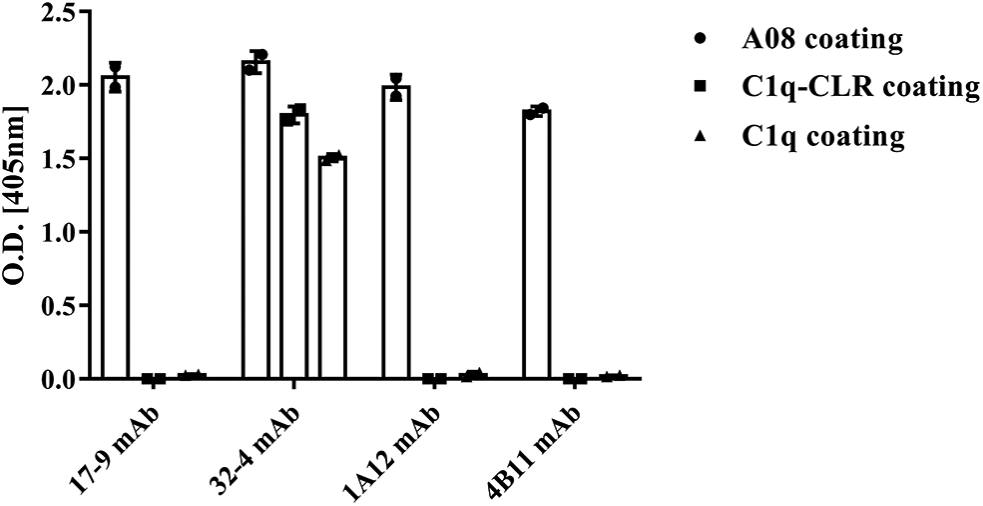
Binding of the four monoclonal anti-C1q A08 antibodies with C1q A08, C1q-CLR, and intact C1q molecules in ELISA. Only 32-4 mAb could bind to plate bound C1q-CLR and intact C1q molecules.

**Figure 3:**
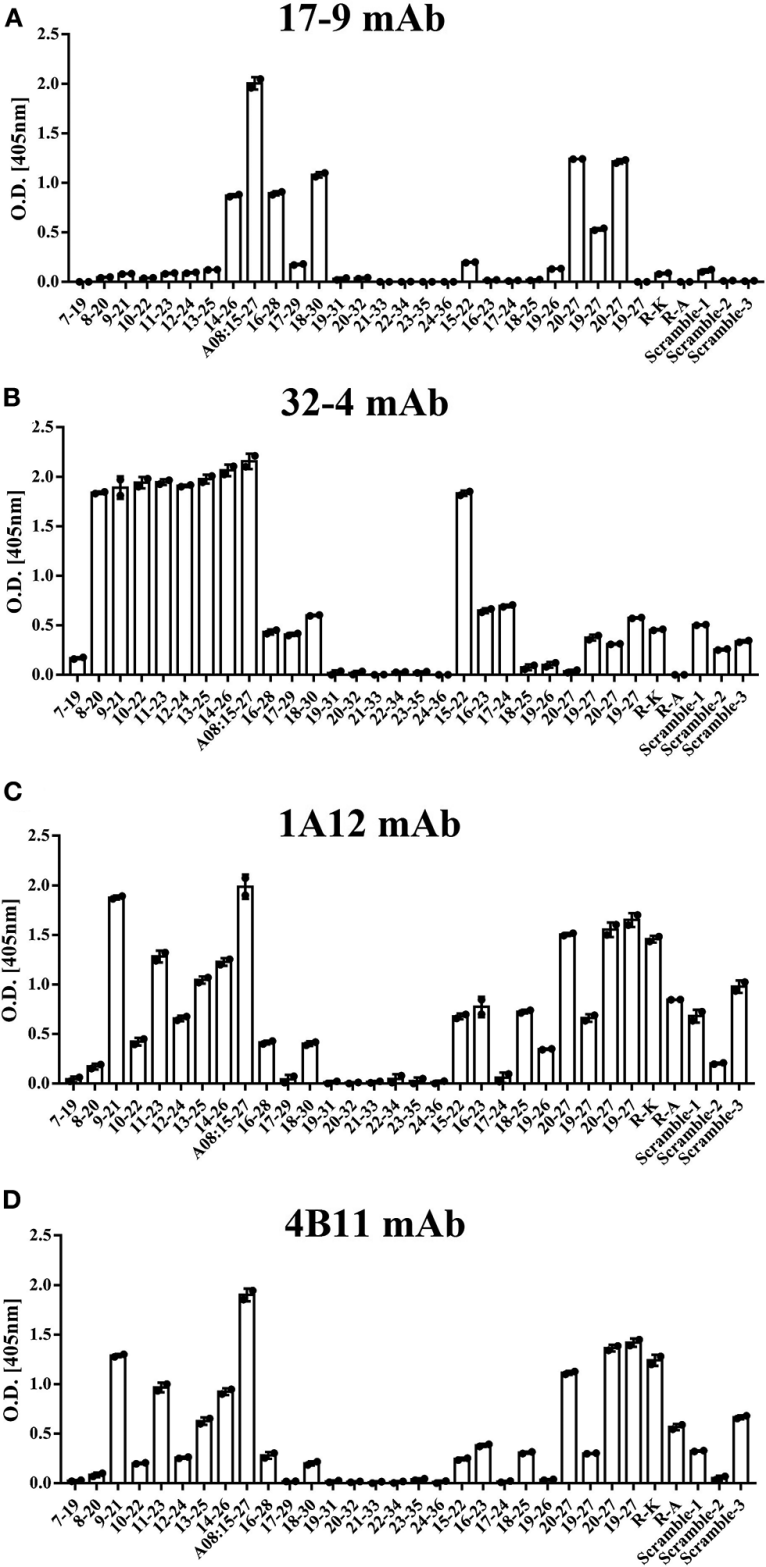
The clones of A08 antibodies. Binding of **(A)** 17-9 mAb. **(B)** 32-4 mAb. **(C)** 1A12 mAb. **(D)** 4B11 mAb with C1q A08-related peptides using ELISA. Eighteen 13-er peptides ranging from 7th to 36th amino acids and six 8-er peptides ranging from 15th to 27th amino acids were synthesized for epitope mapping of different A08 monoclonal antibodies. R-K and R-A, whose arginine was replaced with lysine or alanine, were to verify the role of charge in binding. Three scrambled A08 peptides kept the charge, which disrupted the sequence. It was proposed that 32-4 mAb bond to the six amino acids of N-terminus of C1q A08, while 17-9 mAb bond to eight or 10 amino acids of C-terminus of C1q A08. The binding of 1A12 and 4B11 mAb seemed to depend on the entire sequence of C1q A08. The binding of all the four mAbs to C1q A08 was dependent on both the sequence and charge, as R-K, R-A, and scrambled C1q A08 all showed a much lower binding response.

All the four monoclonal C1q A08 antibodies could bind to C1q A08 peptides using the ELISA (enzyme linked immunosorbent assay) method, while only 32-4 mAb could bind to C1q CLR and intact C1q molecule. Similarly, with anti-C1q A08 antibodies purified from plasma samples of the 10 lupus nephritis patients, 17-9 mAb, 1A12 mAb, and 4B11 mAb did not bind to C1q coated on Costar polystyrene microtiter plates (Figure 2). As illustrated in Figure 3, a group of C1q A08-related peptides were used to map the binding site of the four anti-C1q A08 mAbs on C1q coated on Costar polystyrene microtiter plates. The binding site of 32-4 mAb and 17-9 mAb was on the six N-terminal amino acids and the 10 C-terminal amino acids, respectively, whereas the binding properties of 1A12 and 4B11 were dependent on the entire A08 sequence (Figure 3). The results showed that, when C1q was coated on Costar polystyrene microtiter plates, the six N-terminal amino acids were exposed, but the other seven C-terminal acids were not completely exposed, indicating that A08 was a half-cryptic-half-exposed epitope. A summary diagram of epitope and binding activity for different monoclonal antibodies is shown in Supplementary Figure 1.

### Evaluation of Binding Affinity of C1q and Anti-C1q A08 Antibodies Using BLI

**Figure 4:**
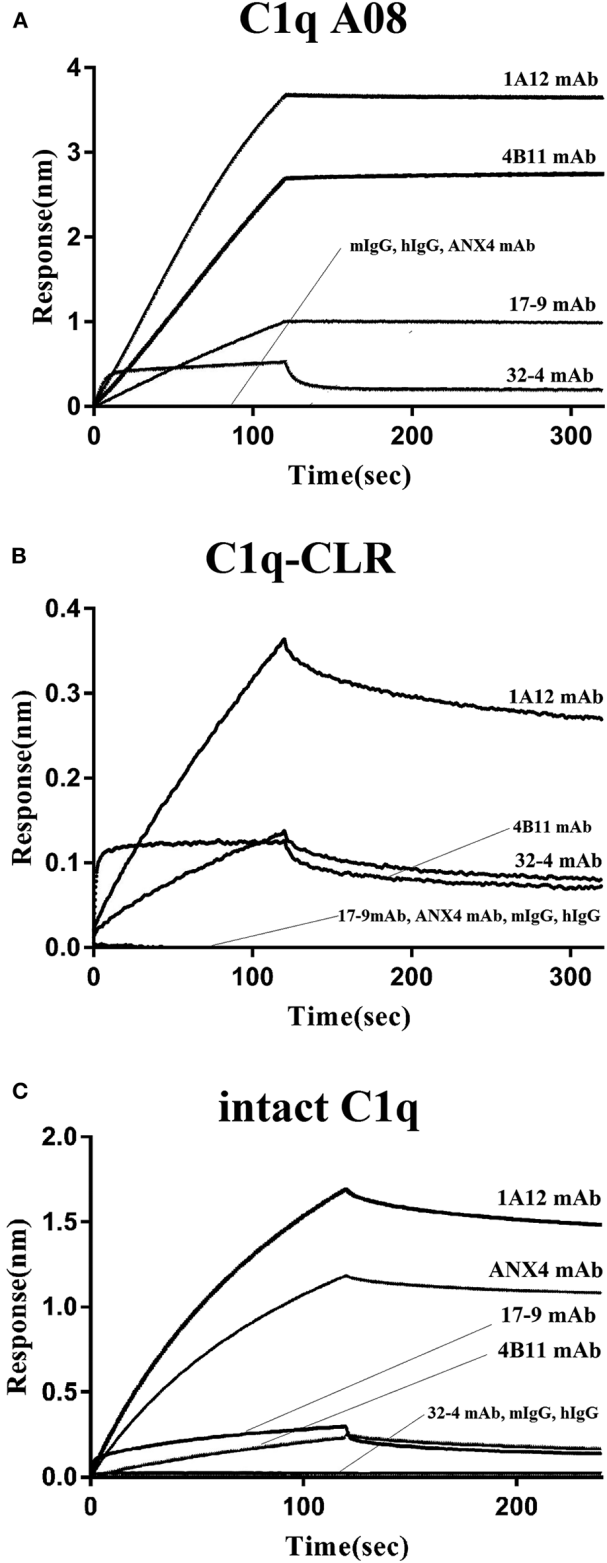
Binding of the four monoclonal anti-C1q A08 antibodies with C1q A08 peptide, C1q-CLR, and intact C1q molecule in BLI. **(A)** SA sensors were coupled with biotin labeled C1q A08 peptide. **(B)** AR2G sensors were coupled with C1q-CLR protein. **(C)** AR2G sensors were coupled with intact C1q molecule.

The binding property of human IgG with C1q was studied to validate if C1q could keep its natural conformation when coupled on an AR2G sensor. *In vivo*, C1q bound to surface bound IgM pentamer or IgG but not free IgM or IgG, which was arranged to clear immune complexes. When human IgG was coupled on an AR2G sensor, C1q could bind to IgG. However, when C1q was coupled on an AR2G sensor, mouse and human total IgG could not bind to C1q at all (Figure 4C).

In the case of the four monoclonal A08 antibodies, 17-9, 1A12, and 4B11, but not 32-4, could bind to C1q and C1qCLR coupled on an AR2G sensor, which was quite opposite to the results in ELISA (Figure 4). The results showed that about 10 C-terminal amino acids were completely exposed but the other three N-terminal acids were not when C1q or C1q CLR was coated on AR2G sensor. Here, A08 was also a half-cryptic epitope but was different from that in ELISA.

### Influence of Monoclonal A08 Antibodies on Activation of Classical Complement Pathway

**Figure 5:**
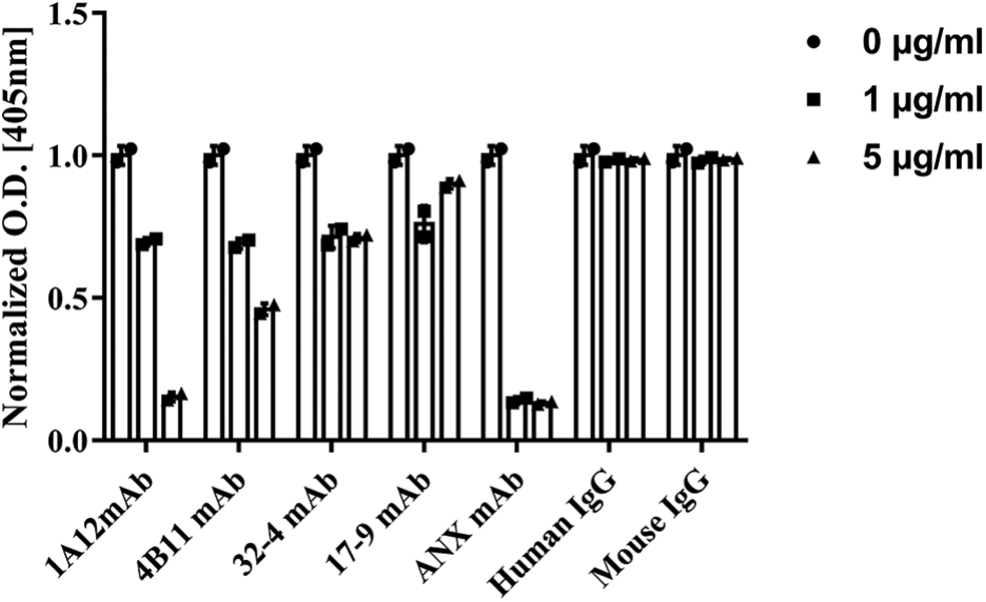
Influence of monoclonal anti-C1q A08 antibodies in activation of classical complement pathway. α3(IV)NC1 was coated on polystyrene microtiter plates and formed immune complex with anti-GBM IgG to activate classical complement pathway. C3b deposition was measured with AP-labeled anti-C3c antibodies, and monoclonal A08 antibodies were mixed with serum to assess their capacity to inhibit the classical complement pathway activation. ANX mAb, a monoclonal antibody targeting the globular region of C1q, was able to inhibit the classical complement pathway activation and was used as a positive control.

The influences of the four monoclonal A08 antibodies on the activation of a classical complement pathway are illustrated in Figure 5. Both 1A12 and 4B11, rather than 17-9 and 32-4, could inhibit the activation of complement classical pathway. The characterization of the four monoclonal anti-C1q A08 antibodies is listed in Table 1. Neither mouse total IgG nor human total IgG influenced the activation of classical complement pathway.

**Table 1:** Characterization of monoclonal anti-C1q A08 antibodies.

**Antibody**	**Biotinylated -C1q A08**	**C1q-CLR**	**C1q**	**Inhibit of C1 activation**
17-9 mAb (mIgG2a)	+	–	–	–
32-4 mAb(hIgG4)	+	+	+	–
1A12 mAb (mIgG3)	+	–	–	+
4B11 mAb (mIgG3)	+	–	–	+
ANX4 mAb (hIgG4)	–	–	+	+
Mouse total IgG	–	–	–	–
Human total IgG			–	–

### Epitope Spreading of C1q After Immunization With C1q A08 *in vivo*

**Figure 6:**
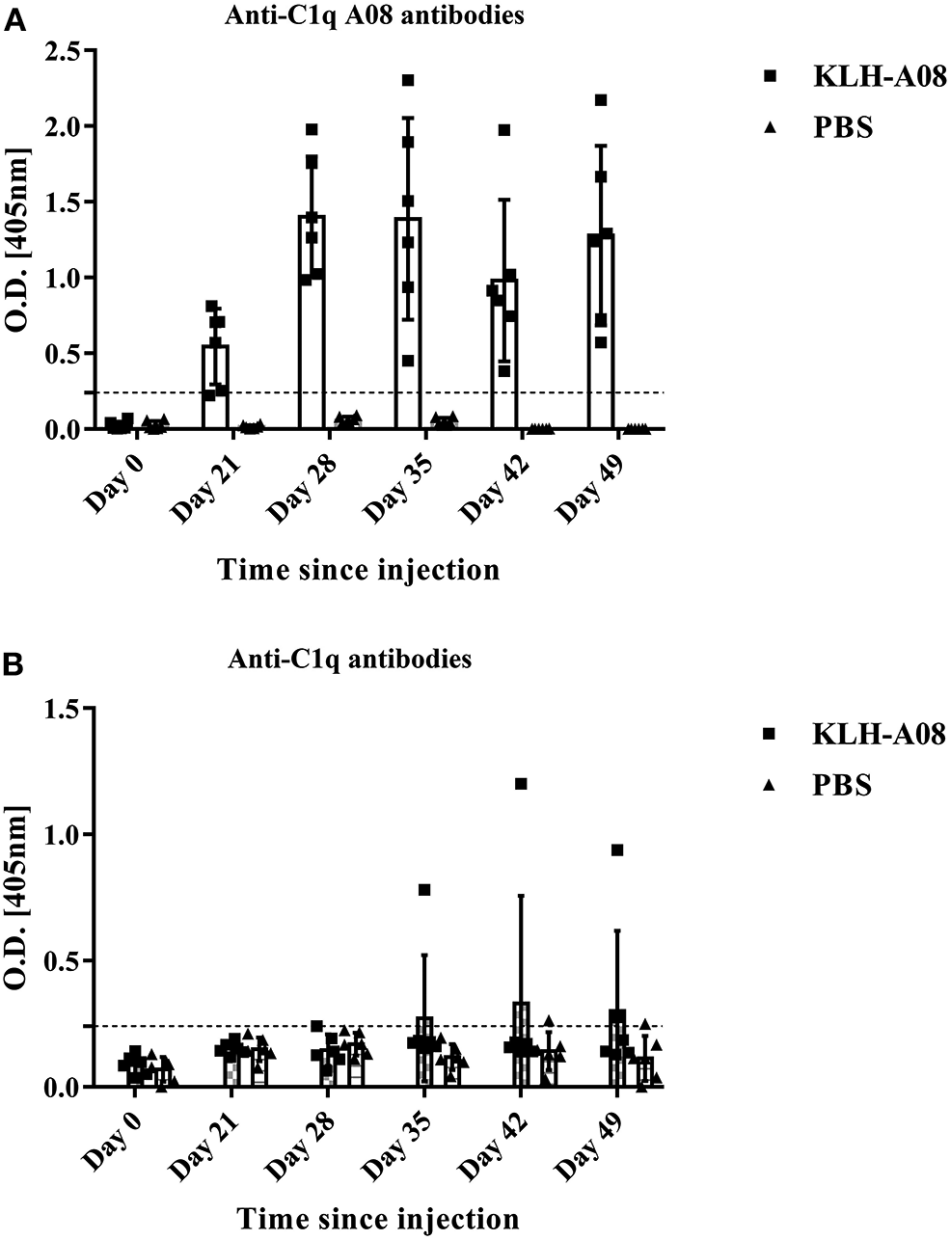
anti-C1q antibodies and anti-C1q A08 antibodies of BALB/C mice immunized with **(A)** KLH-A08 or **(B)** PBS detected using ELISA.

**Figure 7:**
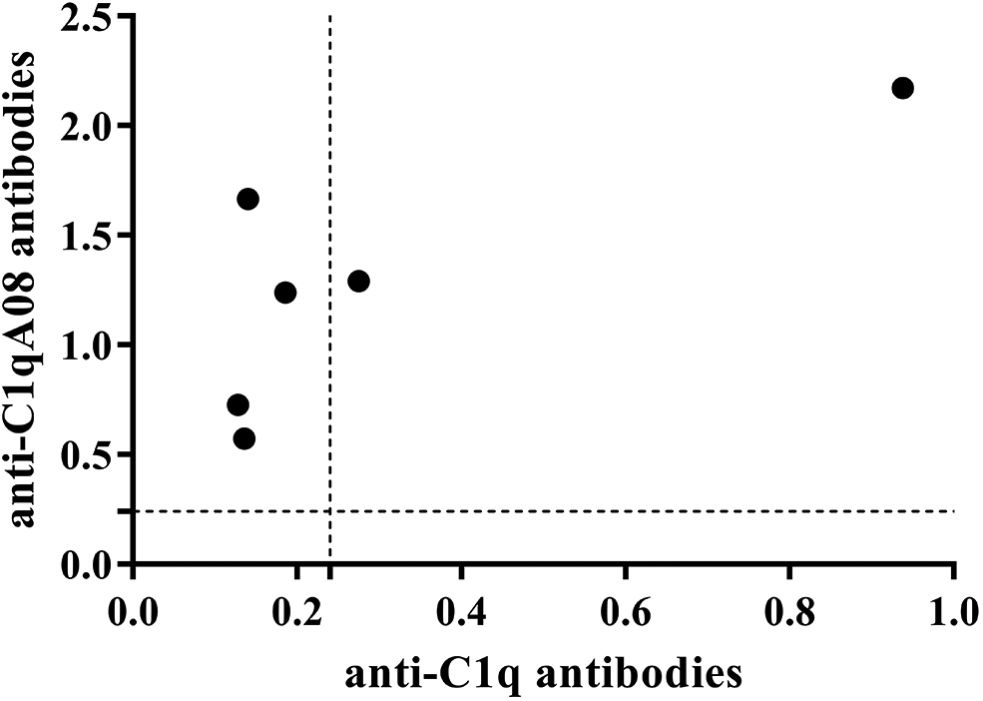
Relationship between C1q antibodies and C1qA08 antibodies of BALB/C mice immunized with KLH-C1q A08 or PBS detected using ELISA.

Anti-C1q A08 antibodies appeared on day 49 in all the six mice of the experimental group but not in the control group (Figure 6A). Furthermore, for the two mice in the experimental group that developed anti-C1q antibodies, one was strongly positive and the other was weakly positive (Figure 6B). The two mice that developed anti-C1q antibodies were also positive for anti-C1q A08 antibodies (Figure 7). The results indicated that epitope spreading of C1q may occur in the two mice with positive anti-C1q antibodies, as anti-C1q A08 antibodies in the other four mice of experimental group did not bind intact C1q by ELISA.

## Discussion

Most of clinical studies indicated that anti-C1q antibodies may be pathogenic in lupus nephritis (23, 24). C1q plays an important role in the clearance of immune complexs and apoptotic cells, whereas anti-C1q antibodies can interfere with the biofunction of C1q. A08 was found to be the most important B cell epitope (12), and the clinical importance of anti-C1q A08 antibodies has been validated in a large Chinese cohort (13). However, the conformational change of C1q A08 in C1q as well as the biofunction of anti-C1q A08 was still unclear.

This study firstly found that all 10 samples from lupus nephritis were positive for anti-C1q A08 antibodies, and seven of them were positive for C1q antibodies. All the anti-C1q A08 antibodies purified from the 10 lupus nephritis patients bound to A08 but not intact C1q coated on ELISA (Figure 1B). The results suggested that A08 peptide amino acids are cryptic or half-cryptic; however, as only 10 samples were included, the possibility cannot be ruled out that some A08 antibodies from lupus patients can bind to intact C1q coated on ELISA. Schaller et al. (25) found that the C1q antibodies of SLE patients are mostly collagen region antibodies, which are merely exposed in the plate-bound C1q. By conducting epitope mapping that utilized one monoclonal antibody of the aforementioned Fabs, Vanhecke et al. (12) identified C1q A08 as the major epitope. When competing with plasma samples to bind to C1q-bound plate, C1q A08 peptides could merely inhibit 10% of binding; but after C1q was degenerated, the inhibition rate of C1q A08 peptides surpassed 30%, suggesting that C1q A08 is a nearly fully-cryptic epitope to which most C1q antibodies did not bind. A BLI assay in which intact C1q was coupled on an AR2G sensor was employed to study the binding property of the four monoclonal A08 antibodies with C1q. It was found that about 10 C-terminal amino acids of the A08 amino acid sequence were completely exposed, but only three N-terminal amino acids were not exposed. Thus, the 17-9 mAb, 1A12 mAb, and 411 mAb, but not the 32-4 mAb, could bind to C1q or C1q CLR in BLI. When C1q was coated on Costar polystyrene microtiter plates, the six N-terminal amino acids were completely exposed, whereas the seven C-terminal amino acids were not exposed. Only the 32-4 mAb, rather than 17-9, 1A12, or 4B11, could bind to C1q in ELISA. Interestingly, all the human anti-A08 antibodies affinity purified from plasma samples of lupus nephritis patients did not bind to the intact C1q by ELISA, implying that the binding property of most A08 autoantibodies from patients are dependent on entire A08 sequences like 1A12 mAb or 4B11 mAb. The sequence of A08 is special with four arginine residues and one lysine residue, which endows C1q A08 with strongly positive charge. It makes sense that such sequences of C1q A08 has strong immunogenicity and nearly all amino acids of A08 are exposed *in vivo*, which makes C1q A08 the most important B-cell epitope. However, when C1q was coated on microtitre plates, the conformation of C1q changed, and several C-terminal amino acids turned cryptic. As a result, nearly all anti-C1q A08 antibodies we unable to bind to C1q in ELISA; 32-4 mAb, however, only recognized the six N-terminal amino acids. Thus, the majority of the anti-C1q A08 antibodies from patients with lupus nephritis could not bind to C1q coated on ELISA plate. That is why anti-C1q A08 antibodies and anti-C1q antibodies of lupus nephritis patients were not all overlapped. Our data showed that the so-called “anti-C1q A08 antibodies” and “anti-C1q antibodies” defined by ELISA were in fact both anti-C1q antibodies but not completely overlapped, which means the so-called “anti-C1q A08 antibodies” are a part of the anti-C1q antibody family, which recognized nearly complete cryptic epitope in ELISA, while the so-called “anti-C1q antibodies” are anti-C1q antibodies that recognized exposed epitope of C1q coated on ELISA plate.

Furthermore, our study showed that C1q A08 is significant in the activation of C1, and, thus, some anti-C1q A08 antibodies can inhibit the complement activation. Though 17-9 mAb, 1A12 mAb, and 4B11 mAb all can bind to C1q or C1q CLR in BLI, only the 1A12 mAb and 4B11 mAb can inhibit the activation of complement classical pathway. The isotypes of 1A12 and 4B11 were mouse IgG3, which can activate the classical complement pathway by Fc, while 32-4 was human IgG4, which cannot activate classical complement pathway. The isotype of 17-9 was mouse mIgG2a, which can activate complement classical pathways. Thus, the isotypes of C1q A08 mAbs seemed to have no influence on activation of complement classical pathway. The influence of anti-C1q A08 antibodies on the activation of C1 depends on the binding site of anti-A08 antibodies on A08. It seemed that the conformation of C1q experiences change during activation, and some anti-C1q A08 antibodies may prevent the process through a stereo-hindrance effect. Those results may provide a novel insight into the process of C1 activation. The complement system plays an important role in the clearance of immune complexes in tissues, and the important pathogenesis involved in lupus nephritis is the dysfunction for the clearance of immune complexes and apoptotic cells. Based on our prior study, this *in vitro* study showed that anti-C1q A08 antibodies may inhibit the activation of complement classical pathway, which may in turn interfere with the clearance of immune complex or apoptotic cells. Moreover, prior studies showed that A08 can bind to the von Willebrand factor (vWF) as well as some other proteins and can activate the complement classical pathway. Thus, anti-C1q A08 antibodies may also interfere with the binding between C1q A08 and other ligands to block the activation of complement classical pathway.

The limitation of the current study mainly lies in the lack of the precise conformational structure of C1q on different surfaces. Antibodies against plate-bound C1q from SLE patients were not isolated and used as controls. The amount of the mouse antibody was too small, and the evidence of epitope spreading was not so solid. Furthermore, anti-C1q A08 antibodies cannot be isolated from the mouse and purified, since the serum sample was not sufficient. Thus, the structural study of C1q is still needed to provide more insights into the role of anti-C1q A08 antibodies in lupus nephritis.

In conclusion, C1q A08, one important but half-cryptic epitope involving six N-terminal amino acids, is important in activation of complement classical pathway, and some anti-A08 antibodies can prevent this process. The epitope spreading of C1q in BALB/C mice immunized with C1q A08 peptide occurred, indicating that C1q A08 is important for development of anti-C1q antibodies detected by ELISA.

## Materials and Methods

### Reagents

Female BALB/C mice were obtained from Beijing Vital River Laboratory Animal Technology Co., Ltd. In the experiments outlined below, avidin from egg white (Aladdin), C1q (EMD chemicals), alkaline phosphatase (AP) substrate P-nitrophenyl phosphate (Sigma-Aldrich), Mouse total IgG (Sigma-Aldrich), and human total IgG (Sigma-Aldrich) were used. Furthermore, the following antibodies were adopted: AP-conjugated polyclonal goat anti-human IgG (γ-chain specific) (Sigma Aldrich), AP-conjugated polyclonal goat anti-mouse IgG (whole molecule, Sigma-Aldrich), AP-conjugated polyclonal goat anti-rabbit IgG (whole molecule, Sigma-Aldrich), and polyclonal rabbit anti-human C3c (Dako). C1q CLR was produced by partial pepsin digestion of C1q as previously described (13).

### Plasma Exchange Samples

Plasma exchange fluids were obtained from anti-C1q A08 antibodies positive lupus nephritis patients during the treatment with plasmapheresis.

Informed consent was obtained for blood and plasma exchange samples. The research was in compliance with the Declaration of Helsinki, and the design of this work was approved by the local ethical committees.

### A08 Monoclonal Antibodies From Hybridoma Cells

Three female BALB/c mice were injected subcutaneously with 0.5 mg A08 peptide coupled with Keyholelimpet hemocyanin (KLH) in complete Freund's adjuvant on day 0, and boosts were performed with 0.5 mg A08 peptide coupled with KLH in incomplete Freund's adjuvant on both day 7 and 14. Splenocytes were fused with the myeloma cell line SP2/0. Fused cells were grown on hypoxanthine-aminopterin-thymidine (HAT) selective semi-solid media for 15 days, and the resulted hybridomas clones were transferred to 96-well tissue culture plates. The supernatants were isolated and tested in an ELISA assay for reactivity against A08 peptides. Positive clones were isotyped and cultured for 30 days to identify stable expressing clones. Four A08 monoclonal antibodies were screened and named as 17-9, 32-4, 1A12, and 4B11, respectively. They were used for binding assays, epitope mapping, BLI, and the complement activation study.

It should be noted that hybridoma cells expressing 32-4 mAb were difficult to culture, and the total mRNA was extracted. After RT-PCR to get cDNA, redundant primers hybridizing to the leader sequence (5′ primer) and to the C region immediately downstream of the V-J region (3′ primer) were used to clone the V regions (26). Cloned V regions of 32-4 mAb were then expressed as joined to the constant region of human IgG4/kappa. The other three monoclonal A08 antibodies 17-9, 1A12, and 4B11 were identified as mouse IgG2a/kappa, mouse IgG3/lambda, and mouse IgG3/lambda, respectively.

### Recombinant Human α3(IV)NC1

The recombinant 6^*^His-tagged human α3(IV)NC1 with signal peptide was cloned into a pcDNA3.1 vector, which was transient transfected with HEK-293T cells. After being cultured for 7 days, the supernatant was collected and applied to HisTrap HP column to isolate human 3(IV)NC1 protein.

### Purification of Total IgG From Plasma Exchange of Anti-GBM Antibody Positive Patients

Total IgG containing antibodies against α3(IV)NC1 was isolated from plasma exchange samples of anti-GBM antibody positive patients using protein G column. Total IgG was eluted with citric acid/sodium citrate buffer (20 mM, pH = 2.7). After adjusting pH to 7.3, the buffer of total IgG was changed to a phosphate-buffered saline (PBS) buffer with 10 kDa ultrafiltration.

The recombinant human α3(IV)NC1 and anti-GBM antibodies were used to form immune complex and to evaluate complement activation via the classic pathway.

### Synthesis of C1q A08 Related Peptides

Biotinylated peptides, non-biotinylated peptides, and KLH-conjugated peptides with >95% purity were synthesized by GenScript, as described previously (13). Peptide A08 (GRPGRRGRPGLKG) is derived from the C1q A chain with sequence from the 15 to 27th amino acids. Eighteen 13-er peptides ranging from the 7 to 24th amino acids and six 8-er peptides ranging from the 15 to 27th amino acids were synthesized for epitope mapping of different A08 monoclonal antibodies. The sequence of all C1q A08 related peptides are listed in Table 2. In parallel, other related peptides (first, scrambled A08, KGGAPRRGGLPRR; second, scrambled A08, RRGPRLRGPKGGG; third, scrambled A08, RPRGLRGPRGGKG; A08 [R → K], GRPGKKGKPGLKG; and A08 [R → A], GRPGAAGAPGLKG) were used as controls (13).

**Table 2:** The sequence of C1q A08-related peptides.

**Sequence**
7–19: PDGKKGEAGRPGR
8–20: DGKKGEAGRPGRR
9–20: GKKGEAGRPGRRG
10–22: KKGEAGRPGRRGR
11–23: KGEAGRPGRRGRP
12–24: GEAGRPGRRGRPG
13–25: EAGRPGRRGRPGL
14–26: AGRPGRRGRPGLK
A08: GRPGRRGRPGLKG
16–28: RPGRRGRPGLKGE
17–29: PGRRGRPGLKGEQ
28–30: GRRGRPGLKGEQG
19–31: RRGRPGLKGEQGE
20–32: RGRPGLKGEQGEP
21–33: GRPGLKGEQGEPG
22–34: RPGLKGEQGEPGA
23–35: PGLKGEQGEPGAP
24–36: GLKGEQGEPGAPG
15–22: GRPGRRGR
16–23: RPGRRGRP
17–24: PGRRGRPG
18–25: GRRGRPGL
19–26: RRGRPGLK
20–27: RGRPGLKG
19–27: RRGRPGLKG
R–K: GRPGKKGKPGLKG
R–A: GRPGAAGAPGLKG
Scramble 1: KGGAPRRGGLPRR
Scramble 2: RRGPRLRGPKGGG
Scramble 3: RPRGLRGPRGGKG

### Isolation and Purification of Anti-C1q A08 Antibodies From Plasma Exchange of Lupus Nephritis Patients

Peptides with >95% purity were synthesized by GenScirpt, and A08-Cys was synthesized where the cysteine was added for conjugation with Pabpur Sulfolink Beads supplied by SMART lifesciences. A carboxyl group of iodoacetic acid was immobilized on resin, and iodine ions are good leaving groups. Thiol of cysteine with low pKa reacts easily with iodoacetic acid to immobilize peptide or protein on resin. About 50 μl of plasma samples from lupus nephritis patients were diluted in a 1:1 ratio with PBS buffer and applied to Protein G column to isolate total IgG, which was eluted with citric acid/sodium citrate buffer (20 mM, pH = 2.7). After adjusting pH to 7.3, total IgG was applied to A08 affinity column to isolate anti-A08 IgG, which was eluted with citric acid/sodium citrate buffer (20 mM, pH = 2.7). After adjusting pH to 7.3, the buffer of anti-A08 IgG was changed to a PBS buffer with 10 kDa ultrafiltration. The purified antibodies were used for testing binding property to plate-bound C1q and C1q A08.

### Detection of Anti-C1q Antibodies, Anti-C1q CLR Antibodies, and Anti-C1q A08 Antibodies With an ELISA Assay

As described previously, (27) human C1q (Sigma-Aldrich), C1q CLR, and neutravidin (Pierce Biotechnology) proteins diluted at previously determined concentrations of 5, 1 μg/ml in 0.05 M bicarbonate buffer (pH = 9.6), and 5 μg /ml in carbonate buffer (0.1 M sodium carbonate, pH = 9.6) were coated on the wells of one half of polystyrene microtiter plates (Costar, Corning) at 4°C overnight, respectively. The wells in the other half were coated with bicarbonate or carbonate buffers alone to act as antigen-free wells. Free binding sites were blocked with 0.01 M PBS containing 0.1% Tween 20 (PBST) and 1% (10 mg/ml) bovine serum albumin except for the neutravidin plates at 37°C for 1 h, which were incubated with biotinylated peptides at 5 mg/ml in PBS at room temperature for 2 h. Sera were diluted to 1:200 in PBST/0.5 M NaCl to detect anti-C1q and anti-C1q CLR antibodies, and 1:200 in 0.1% PBST to detect anti-A08 related peptides antibodies. The volumes for both this step and subsequent steps were 100 μl, and all incubations were carried out at 37°C for 1 h. The plates were washed three times with PBST. Alkaline phosphatase–conjugated anti-human IgG (Calbiochem), diluted at 1/3,000, was used as a detection antibody. The P-nitrophenyl phosphate (1 mg/ml; Sigma-Aldrich) was used in substrate buffer (1.0 M diethanolamine and 0.5 mM MgCl_2_, pH = 9.8). Optical density was measured at 405 nm. Samples were considered positive if they exceeded the mean plus 2 SD from 100 healthy blood donors.

### Biolayer Interferometry (BLI) Assays

#### Octet Binding Assay of C1q, C1q A08, and C1q CLR to Anti-C1q A08 Antibodies

SA sensors were used to load biotinylated A08 peptides at a concentration of 20 μg/mL, and the loading level was about 1.5 nm. The sensors were then moved to HEPES (hydroxyethyl piperazine ethanesulfonic acid) buffer wells for baseline generation and subsequently to anti-C1q A08 antibodies wells (100 nM) for 120 s. Then they were dipped to HEPES buffer wells for dissociation for 200 s. The sensors were generated with glycine-HCl buffer (100 mM, pH = 2.5). ANX4 mAb, normal mouse and human total IgG were used as controls.

AR2G sensors were used to load C1q CLR and C1q at a concentration of 10 μg/mL, and the loading level was about 2.5 nm. The sensors were then moved to HEPES buffer wells for baseline generation and subsequently to anti-C1q A08 antibodies wells (100 nM) for 120 s. They were then dipped into HEPES buffer wells for dissociation for 200s. The sensors were generated with glycine-HCl buffer (100 mM, pH = 2.5). ANX4 mAb, normal mouse, and human total IgG were used as controls.

### C3 Activation Assay

Detection of C3 activation was carried out as previously described with brief modification (22). Polystyrene microtiter plate (Costar) was coated with 100 μl of 10 μg/mL recombinant human α3(IV)NC1 in 0.05 M bicarbonate buffer. After overnight incubation, the wells were blocked with 0.2% (w/v) gelatin in PBST and then washed with PBST. IgG from anti-GBM antibody-positive patients was diluted to 200 μg/ml in PBST and added to the plates. ANX4 mAb, which is an antibody targeting the globular region of C1q that can also inhibit activation of the complement classical pathway, was used as positive control (28), while normal mouse and human total IgG were used as negative controls. After incubation and washing, the mixture of serum samples and anti-A08 antibodies samples was diluted in VBS [5 mM barbital, 145 mM NaCl, 0.15 mM CaCl_2_, 1 mM MgCl_2_, and 0.1% Tween 20, pH = 7.4], added to the plates, and incubated at 37°C for 1 h. The plates were washed with VBST, and the bound C3b was detected using rabbit anti-human C3c (Dako), followed by alkaline phosphatase-conjugated goat anti-rabbit IgG (Sigma Aldrich); this was then followed by the colorimetric substrate, P-nitrophenyl phosphate (1 mg/ml; Sigma-Aldrich). The results were recorded as the net optical absorbance (average value of antigen wells minus average value of antigen-free wells) at 405 nm in an ELISA reader (Bio-Rad 550).

### Immunization With C1q A08 Peptide

Twelve 5-week-old female BALB/C mice were divided into two groups. For experimental group, six mice were immunized subcutaneously with 0.5 mg KLH-conjugated C1q A08 peptide in incomplete Freund's adjuvant (IFA, Difco Laboratories) on day 0, followed by subcutaneous booster injections of 0.5 mg KLH-conjugated C1q A08 peptide in complete Freund's adjuvant (CFA, Difco Laboratories) on days 7 and 14. For control group, six mice were immunized with PBS buffer only in adjuvant using the identical schedule. Sera was obtained on days 0, 21, 28, 35, 42, and 49, respectively. Anti-A08 antibodies and anti-C1q antibodies were detected by ELISA, and alkaline phosphatase-conjugated goat F(ab')^2^ anti-mouse IgG (Abcam) was used as secondary antibody.

### Statistical Analyses

Differences of quantitative parameters between groups were assessed using the *t*-test for data normally distributed or the non-parametric test for data not normally distributed. Differences of semi-quantitative data were tested using the Mann–Whitney *U*-test. Differences of qualitative data were compared using the χ^2^ test. The Spearman Correlation was used to analyze the correlation. Analyses were performed with statistical software SPSS 21.0. *p* < 0.05 was considered as significant.

## Data Availability Statement

The raw data supporting the conclusions of this article will be made available by the authors, without undue reservation, to any qualified researcher.

## Ethics Statement

The studies involving human participants were reviewed and approved by Ethical committee of Peking University First Hospital. The patients/participants provided their written informed consent to participate in this study. The animal study was reviewed and approved by Ethical committee of Peking University First Hospital.

## Author's Note

C1q plays an essential role in the adaptive and innate immune system, and C1q A08 is an important epitope of C1q in lupus nephritis patients. In this paper, it was confirmed that C1q A08 is a half-cryptic epitope using the ELISA method, indicating that most anti-C1q A08 antibodies from lupus nephritis patients could not bind to C1q with ELISA. Further, it was found that C1q A08 plays an important role in activation of complement classical pathway. Moreover, it was revealed that epitope spreading of C1q occurred in the mice immunized with C1q A08 peptides. Our findings demonstrated the relationship between anti-C1q antibodies and anti-C1q A08 antibodies, implying that C1q A08 may play an important role in pathogenesis of lupus nephritis.

## Author Contributions

M-HZ, FY, W-JW, and YT designed the study, analyzed the data, and wrote the manuscript. X-LL collected the samples.

## Conflict of Interest

The authors declare that the research was conducted in the absence of any commercial or financial relationships that could be construed as a potential conflict of interest.

## Supplementary Material

Supplementary Figure 1 32-4 mAb bond to the six amino acids of N-terminus of C1q A08, while 17-9 mAb bond to eight or 10 amino acids of C-terminus of C1q A08. The binding of 1A12 and 4B11 mAb seemed to depend on the entire sequence of C1q A08. 1A12 mAb and 4B11 mAb could bind to the 10 amino acids of C–terminal of C1q A08, while the three amino acids of N-terminal of C1q A08 also contributed to the binding.
