# Supplementary figures and images for: C1q A08 Is a Half-Cryptic Epitope of Anti-C1q A08 Antibodies in Lupus Nephritis and Important for the Activation of Complement Classical Pathway
Source: Front Immunol. 2020 May 27;11:848. doi: 10.3389/fimmu.2020.00848 (PMC7267003; doi:10.3389/fimmu.2020.00848)

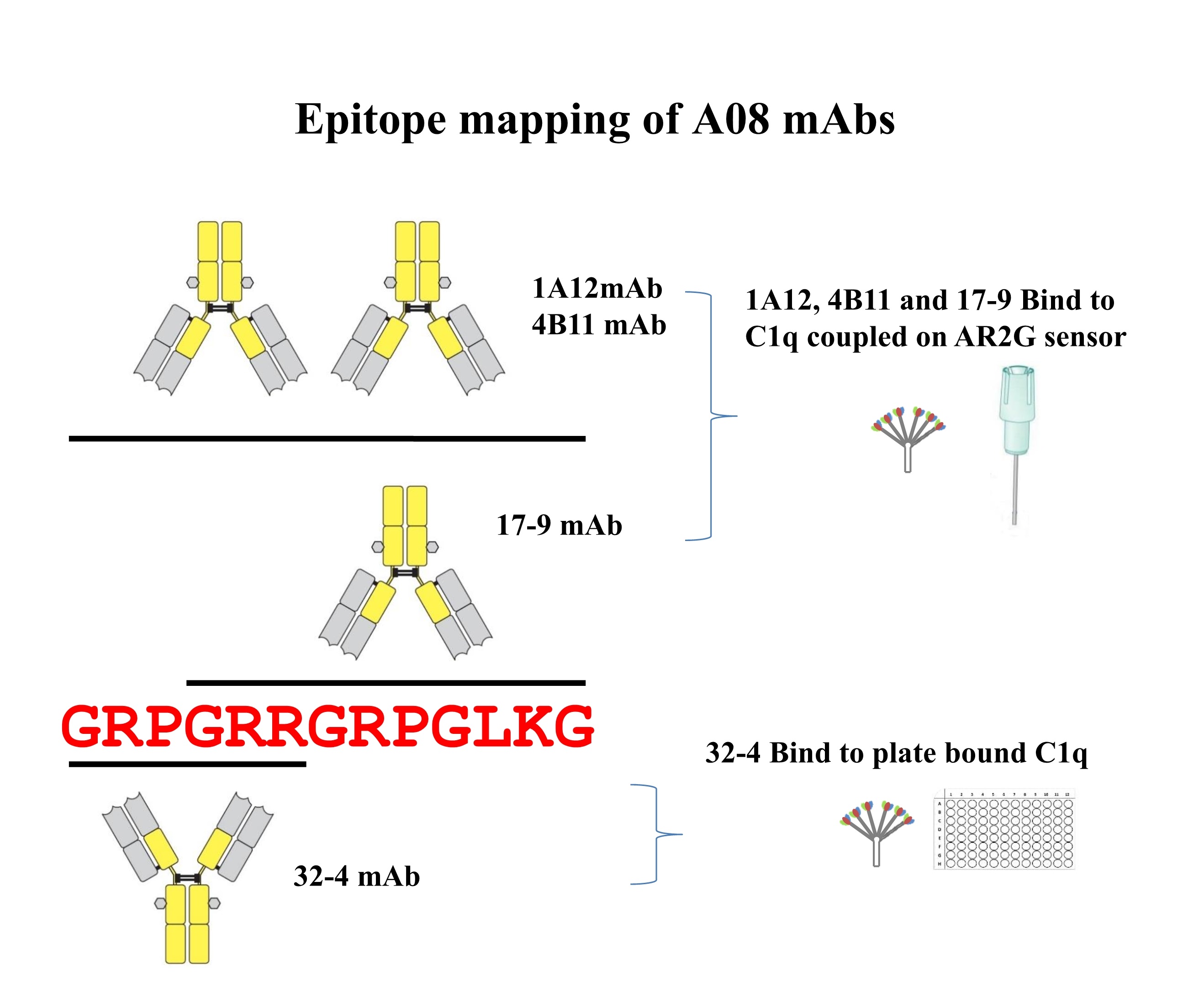

Supplement: Supplementary Figure 1 — 32-4 mAb bond to the six amino acids of N-terminus of C1q A08, while 17-9 mAb bond to eight or 10 amino acids of C-terminus of C1q A08. The binding of 1A12 and 4B11 mAb seemed to depend on the entire sequence of C1q A08. 1A12 mAb and 4B11 mAb could bind to the 10 amino acids of C–terminal of C1q A08, while the three amino acids of N-terminal of C1q A08 also contributed to the binding. [file Image_1.JPEG]
